# Micro‐Focused Ultrasound Treatment for Periocular Skin Leading to Corneal Leukoma With Iridocyclitis: A Case Report

**DOI:** 10.1111/jocd.70601

**Published:** 2025-12-10

**Authors:** Jie Liu, Hongmei Luo, Ni Li

**Affiliations:** ^1^ Department of Ophthalmology West China Hospital, Sichuan University Chengdu China; ^2^ West China School of Nursing Sichuan University Chengdu China

**Keywords:** corneal leukoma, iridocyclitis, micro‐focused ultrasound

## Abstract

**Background:**

Micro‐focused ultrasound (MFUS) converts the mechanical energy of high‐frequency vibrations into thermal energy and induces collagen remodeling through thermal effects to achieve skin tightening and lifting. However, when employed for periocular skin treatment, MFUS carries a potential risk of ocular injury, which deserves high attention from cosmetic surgeons.

**Aims:**

This article reports a case of a young female patient who developed transient visual acuity decline with iridocyclitis and residual corneal leukoma after MFUS treatment on the periocular skin.

**Patient:**

A 32‐year‐old female patient underwent facial MFUS treatment at a cosmetic clinic to improve facial skin laxity. During the treatment of the right periocular skin, the patient developed right eye pain and visual acuity decline. The patient was treated with nonsteroidal anti‐inflammatory drugs (NSAIDs) and glucocorticoids in the right eye and followed up for 3 months. The visual acuity of the right eye improved, but a permanent corneal leukoma persisted.

**Results:**

While MFUS treatment targeting the periorbital area can improve skin laxity, it poses a risk of corneal injury and iridocyclitis. After the treatment, the iridocyclitis resolved, but a permanent corneal leukoma remained.

**Conclusions:**

Periocular MFUS treatment carries the risk of irreversible thermal damage to ocular tissues. Cosmetic surgeons must strictly determine the intensity threshold for periocular MFUS and ensure proper application of eye‐protective devices.

## 1 Introduction

Micro‐focused ultrasound (MFUS) is widely employed in skin rejuvenation and tightening treatments. The Ultraformer III (CLASSYS, South Korea) precisely delivers thermal energy (above 333.15 K) to the dermis and superficial musculo‐aponeurotic system (SMAS) through focused ultrasound, triggering collagen remodeling to achieve skin tightening and lifting [1, 2]. Commonly reported complications following MFUS treatment include facial skin edema, erythema, and transient motor and sensory nerve abnormalities [3, 4]. These complications typically resolve spontaneously over time. However, the risk of ocular tissue damage during MFUS treatment on the periocular skin has been underestimated. Currently, clinical cases have been reported where periocular MFUS treatment led to symptoms such as corneal leukoma, iris atrophy, and lens opacification [5, 6, 7]. We present a case of a 32‐year‐old female patient who developed transient visual acuity loss with iridocyclitis during periocular MFUS (Ultraformer III) treatment. Following 3 months of treatment, her vision recovered and iridocyclitis resolved, though a corneal leukoma persisted.

## 2 Case Report

This article reports a case of a 32‐year‐old female patient who developed ocular complications during periocular skin MFUS treatment and was followed up for 3 months. A 32‐year‐old female patient sought to improve facial skin laxity and underwent facial MFUS treatment at a medical aesthetic clinic. During the procedure, no eye shields were worn. When undergoing treatment of the right periocular skin, the patient developed right eye pain, lacrimation, and decreased visual acuity. Following the onset of right eye pain, treatment was discontinued, and the patient sought evaluation at a local hospital. Since the patient first visited an external hospital, except for the patient's self‐reported normal intraocular pressure, the visual acuity and ocular physical examination data were incomplete. The local hospital prescribed the following medications for the right eye: dexamethasone‐tobramycin eye drops (four times daily), deproteinized calf blood extract eye gel (four times daily), and sodium hyaluronate eye drops (four times daily). The patient reported that after medication administration, her right eye visual acuity had improved compared with that before treatment, but the symptom of blurred vision still persisted. Fourteen days later, the patient was using only sodium hyaluronate eye drops (four times daily) in the right eye, with no improvement in blurred vision.

**FIGURE 1:**
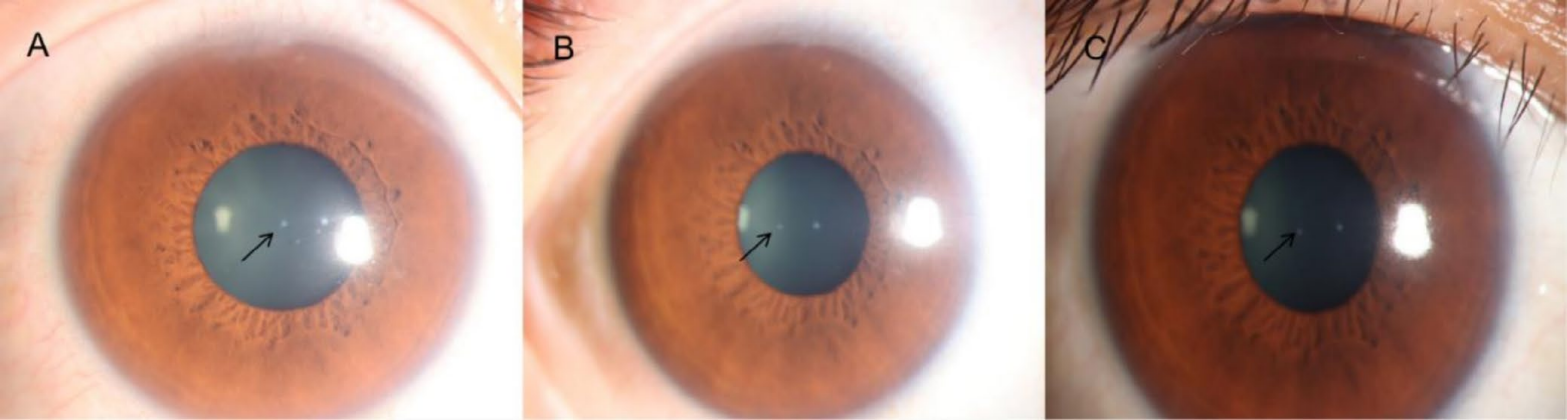
(A) Corneal opacity at 36 days after MFUS treatment (black arrow); (B) Corneal opacity at 62 days after MFUS treatment; (C) Corneal opacity at 95 days after MFUS treatment.

**FIGURE 2:**
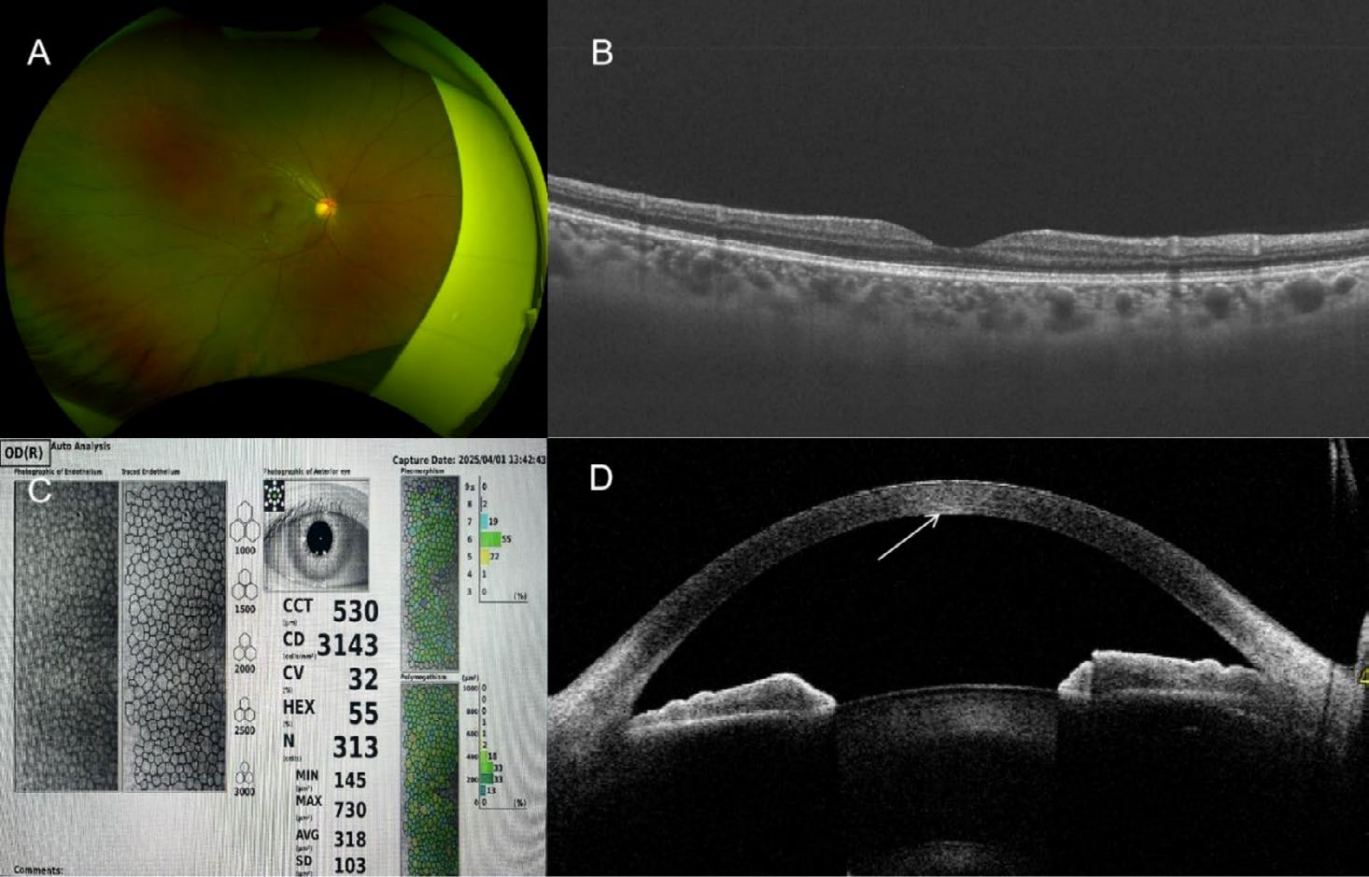
(A) No abnormalities were found in the patient's retina; (B) No abnormalities were detected in the patient's macula; (C) The patient's corneal endothelial examination was normal; (D) Opacities were visible in the patient's corneal stroma (white arrow).

Thirty‐six days later, the patient visited the ophthalmology department of our hospital. The Snellen visual acuity was 20/20 in the right eye and 20/16 in the left eye, with intraocular pressures of 16.1 mmHg in the right eye and 14.0 mmHg in the left eye. Ophthalmic examination of the right eye revealed two punctate opacities involving the deep stromal layer in the central corneal pupillary zone, with two separate punctate opacities affecting the superficial stromal layer noted in the non‐pupillary region at the 5 o'clock and 8 o'clock positions. The remaining corneal stroma was transparent (Figure 1A). Keratic precipitates (KP) were present on the corneal endothelium, and anterior chamber cells were observed. Anterior chamber depth was normal, iris texture was unremarkable, pupil was round with brisk light reflex, lens was transparent, and fundus examination showed no abnormalities (Figure 2A,B). Ophthalmic examination of the left eye showed no abnormalities. Corneal endothelial microscopy of the right eye showed no abnormalities (Figure 2C). Detailed inquiry was made regarding the patient's treatment. The treatment was performed on the lateral third of the upper eyelid. A 2.0‐mm probe was applied at 0.4 J per pulse to the periocular skin, with a total of 36 shots delivered. The patient had an 8‐year history of right eye Posner–Schlossman syndrome (PSS), with an average of 2–3 relapses per year. The patient was diagnosed with right corneal leukoma and right iridocyclitis in our hospital. She was prescribed pranoprofen eye drops (twice‐daily) and prednisolone acetate eye drops (four times daily) for the right eye. At the 62‐day follow‐up visit, the patient reported improvement in blurred vision. The Snellen visual acuity was 20/16 in the right eye and 20/16 in the left eye, with intraocular pressures of 10.0 mmHg in the right eye and 16.2 mmHg in the left eye. Ophthalmic examination of the right eye revealed two punctate opacities that were still present in the central pupillary zone of the cornea, involving the deep stromal layer. Two punctate opacities were noted in the non‐pupillary area at the 5 o'clock and 8 o'clock positions, involving the superficial stromal layer. The remaining cornea was transparent (Figure 1B). KPs were absent, and there was no evidence of anterior chamber cells. The remaining ophthalmic examination findings were unremarkable. Additionally, the examination of the left eye revealed no abnormalities. The medication for the patient's right eye was adjusted to pranoprofen eye drops (twice‐daily) and sodium hyaluronate eye drops (four times daily). At the 95‐day follow‐up visit, the Snellen visual acuity was 20/16 in the right eye and 20/16 in the left eye, with intraocular pressures of 10.9 mmHg in the right eye and 13.4 mmHg in the left eye. Ophthalmic examination revealed two persistent opacities involving the deep stromal layer in the central pupillary zone of the right cornea, with two stable punctate opacities in the non‐pupillary region at the 5 o'clock and 8 o'clock positions (unchanged from prior findings). The remaining ocular structures were unremarkable, and the left eye showed no abnormalities (Figure 1C). Anterior segment optical coherence tomography of the right eye confirmed corneal stromal opacities (Figure 2D).

## 3 Discussion

Ultrasound refers to an oscillating sound pressure wave that causes high‐frequency vibration of target tissue molecules, converting mechanical energy into thermal energy. The principle of MFUS for skin treatment is that ultrasound can precisely heat the target tissue to above 333.15 K, generating a region of coagulative necrosis. This process disrupts the cell membranes of the deep dermis and SMAS. Following cell apoptosis and necrosis, wound healing activates relevant cascading reactions, stimulating the differentiation of fibroblasts and the production of new collagen. Skin rejuvenation and tightening are achieved through the remodeling of dermal collagen [8].

Ultraformer III, developed and manufactured by CLASSYS, a listed Korean company, is equipped with seven different functional probes. For facial skin applications, the commonly used probes are 3.0 and 4.5 mm. The 3.0‐mm probe can reach the deep fat layer during treatment, enhancing the tightness of the mid‐facial skin. The 4.0‐mm probe, meanwhile, can access the fascial layer, effectively improving nasolabial folds and neck wrinkles. In clinical practice, these two probes are generally combined for facial skin treatment. Compared with facial skin, the periorbital skin has thinner epidermis, thinner dermis, and less subcutaneous fat; thus, ultrasonic energy easily acts directly on the orbicularis oculi muscle, orbital septum, and even reaches ocular tissues. In addition, the ocular structure is complex: The periorbital region has abrupt multilayer structural transitions (skin‐orbital bone‐eyeball), leading to irregular energy attenuation during penetration. Ultrasonic energy also tends to reflect off the orbital bone surface, causing focal point deviation. In contrast, facial tissue layers are distinct, with stable ultrasonic energy attenuation, allowing the focal point to be accurately localized at the preset depth.

MFUS causes corneal stromal opacification because the ultrasonic thermal energy induces denaturation of collagen in the corneal stroma and disrupts the orderly arrangement of collagen fibers, leading to the formation of scarring opacification. In addition, damaged corneal tissue can release inflammatory cytokines, triggering corneal stromal inflammation that leads to corneal opacification, edema, and decreased transparency. The iris and ciliary body are uveal tissues, rich in blood vessels and nerves, and are highly sensitive to inflammatory stimuli. The patient's iridocyclitis may be primarily attributed to the direct stimulation of the blood vessels and nerves within the iris and ciliary body by ultrasonic energy. This stimulation causes vasodilation and increased vascular permeability, resulting in serous exudation. Additionally, the release of cellular debris and antigenic substances from damaged tissues activates the immune system, triggering an autoimmune reaction, which further exacerbates the inflammatory process in the uveal tissues. The patient has a history of PSS with 2–3 annual relapses, which may predispose them to more readily triggering autoimmune reactions following ultrasonic energy stimulation. It is important to note that this report has inherent limitations, given that the patient initially presented to another institution rather than our hospital, leading to incomplete baseline ophthalmological data.

## 4 Conclusion

MFUS treatment can effectively improve skin laxity and wrinkles to achieve anti‐aging effects. However, improper treatment of the periorbital skin may trigger severe ocular diseases. This article reports a case of decreased visual acuity, corneal leukoma, and iridocyclitis following MFUS facial rejuvenation treatment. Following treatment, the patient's visual acuity improved and iridocyclitis resolved, though the corneal leukoma remained irreversible. Aesthetic physicians must maintain a high degree of vigilance regarding the risk of ocular tissue injury during periorbital procedures. We advocate avoiding MFUS treatment in the periorbital region. If treatment in the eye area is necessary, protective eye shields should be used. Beyond this, MFUS for periorbital skin requires a low‐depth probe and low‐energy settings. Such procedures should be performed by experienced and skilled aesthetic physicians.

## Author Contributions

**Jie Liu:** responsible for case collection, data collation, and initial manuscript drafting. **Hongmei Luo, Ni Li:** involved in clinical diagnosis, formulation of treatment plans, manuscript revision and final manuscript review.

## Funding

The authors have nothing to report.

## Ethics Statement

The authors have nothing to report.

## Consent

Written informed consent for the publication of this case report, including clinical details and any potentially identifying information, was obtained from the patient. All personal identifiers have been removed or anonymized to protect patient confidentiality. The original consent form is available for review upon request by the journal.

## Conflicts of Interest

The authors declare no conflicts of interest.

## Data Availability

The data that support the findings of this study are available from the corresponding author upon reasonable request.
